# Assembly of MXene/PP Separator and Its Enhancement for Ni-Rich LiNi_0.8_Co_0.1_Mn_0.1_O_2_ Electrochemical Performance

**DOI:** 10.3390/polym12102192

**Published:** 2020-09-25

**Authors:** Qiu-Shi Rao, Song-Yi Liao, Xing-Wen Huang, Yue-Zhu Li, Yi-Dong Liu, Yong-Gang Min

**Affiliations:** 1 School of Materials and Energy, Guangdong University of Technology, Guangzhou 510006, China; R253946192@163.com (Q.-S.R.); hxw1015155619@163.com (X.-W.H.); yuezhu1008@163.com (Y.-Z.L.); ydliu@gdut.edu.cn (Y.-D.L.); 2 Dongguan South China Design Innovation Institute, Dongguan 523808, China

**Keywords:** polypropylene composite membrane, MXene, Ni-rich LiNi_0.8_Co_0.1_Mn_0.1_O_2_, electrochemical performance

## Abstract

In this work, a few-layer MXene is prepared and sprinkled on a commercial polypropylene (PP) separator by a facile spraying method to enhance the electrochemistry of the Ni-rich LiNi_0.8_Co_0.1_Mn_0.1_O_2_ (NCM811) cathode. Scanning electron microscope (SEM) and X-ray diffraction (XRD) are used to characterize the morphology and structure of MXene. Fourier transform infrared spectroscopy (FT-IR) and a contact angle tester are used to measure the bond structure and surface wettability PP and MXene/PP separator. The effect of the MXene/PP separator on the electrochemical performance of ternary NCM811 material is tested by an electrochemical workstation. The results show that the two-dimensional MXene material could improve the wettability of the separator to the electrolyte and greatly enhance the electrochemical properties of the NCM811 cathode. During 0.5 C current density cycling, the Li/NCM811 cell with MXene/PP separator remains at 166.2 mAh/g after the 100 cycles with ~90.7% retention. The R_ct_ of MXene/PP cell is measured to be ~28.0 Ω. Combining all analyses results related to MXene/PP separator, the strategy by spraying the MXene on commercial PP is considered as a simple, convenient, and effective way to improve the electrochemical performance of the Ni-rich NCM811 cathode and it is expected to achieve large-scale in high-performance lithium-ion batteries in the near future.

## 1. Introduction

With the development of electric vehicles in recent years, the power sources of lithium-ion batteries (LIBs) are receiving higher demands for improved energy density, charging rate, and cycle performance [1,2,3,4,5]. Therefore, Ni-rich cathodes with higher capacity are currently being accelerated to be used in LIBs. Specially, LiNi_0.8_Co_0.1_Mn_0.1_O_2_ (NCM811) is attracting increasing attention due to its specific capacity of ~200 mAh/g [6,7,8]. However, its poor mechanical strength, cycling stability, and rate capability impose limitations on its large-scale practical application [9]. In order to solve these problems, most strategies are focusing on the modification of NCM811 [8,9,10,11,12]. However, the modification process is time-consuming, labor-intensive, and energy-intensive, and it is difficult to achieve mass production. Therefore, it is very necessary to find a more efficient and facile method to improve the electrochemical performance of Ni-rich NCM811 material [8,10,13].

According to previous work [14,15,16,17], the LIBs performances can be greatly improved by utilizing the modified separator which plays a decisive role in the resistance, capacity, and safety. Therefore, it seems that modifying the separator may be a relatively simple way to improve the performance of the NCM811 battery. For examples, Cho et al. [14] has synthesized amino-functionalized silica particles which were coated on both sides of a polyethylene (PE) film to improve the electrolyte’s wettability for the separator, stability, and cycle performance of battery. Song et al. [15] applied polyimide to a polyethylene film by a simple dipping method, which reduced the resistance by heating shrinkage at high temperatures without affecting the electrochemical performance. An et al. [16] has coated alumina nanopowder and electrospun PVDF nanofibers on both sides of the polyethylene separator. Moreover, due to the special characteristics of two-dimensional materials, numerous pioneering reports are associating with the modified separators with transient metal-based 2D structures. Zhang et al. [18] anchored a new type of 2D/1D V_2_O_5_ nanoplate to the carbon nanofiber (V-CF) middle layer of a PP separator to enhance the electrochemical stability of lithium-sulfur batteries. Li et al. [19] studied functional porous bilayer composite separators for application in the high-current density lithium metal anodes. Chen et al. [20] prepared two-dimensional (2D) molybdenum nitride nanosheets modified Celgard membrane to improve the electrochemical performance of Li-S battery Moreover, as a novel two-dimensional material, the M_n+1_X_n_T_x_ materials (MXene) also attract increasing attentions in electrochemical, adsorption, catalysis, and water treatment [21,22,23,24]. Considering the functional groups (–O, –F, –OH etc.) on the surface of MXene [21,23,24,25,26,27,28,29,30,31,32,33], Song et al. immobilized MXene-functionalized separators to improve electric conductivity and the effective trapping of polysulfides in Li–S battery [34]. The performance of batteries assembled by using modified separator has been greatly enhanced in different aspects.

However, most of the reported works related to the two-dimensional material-based hybrid separator are mainly focused on the immobilization of polysulfides for stable lithium-sulfur batteries [35]. The modified membranes rarely involve improving the electrochemical performance of the currently practical nickel-rich NCM811, such as its safety and stability concerns. Recently, we found that the few-layer and rod-like MXene (Ti_2_C_3_T_x_) as a multifunctional additive can significantly enhance the mechanical properties, cycling performance, and rate capability of Li/NCM811 cells [36]. Therefore, taking into account the important position of the separator in LIBs and previous work, we believe that the current dilemma of NCM811 may be solved by modifying the commercial PP separator with MXene load. In addition, the modification of the separator with MXene will also be a relatively simple, cheap, and easy-to-obtain strategy to enhance the performance of LIBs, which can be quickly and large-scale applied in the current NCM811-based high-energy density battery.

In this work, the objective is to prepare the MXene-modified commercial Celgard PP separator (shorten as MXene/PP) by using simple spraying method. The structure, composition, and morphology of MXene/PP will be investigated by XRD, FT-IR, and SEM, respectively. The effects of MXene/PP on the mechanical and electrochemical performances of NCM811 cathode will be analyzed in details. As a synergistic effect of structural limitation and chemical adsorption, the negatively charged MXene can effectively inhibit the migration of high-valent metal (Ni/Co/Mn) ions in nickel-rich NCM811 to the negative electrode. In addition, MXene has excellent electronic conductivity similar to metals, which enhances the interfacial charge transfer. With these advantageous features, the NCM811/Li battery using MXene/PP separator exhibits excellent electrochemical performance.

## 2. Experimental Section

### 2.1. Chemicals and Materials

All reagents including lithium fluoride (LiF, Aladdin, AR), hydrochloric acid (HCl, Sinopharm, 12 mol/L), Ti_3_AlC_2_ (MAX, Laizhou kaiene ceramic material, 99.8%), tetrabutylammonium hydroxide (TBAOH, Aladdin, AR, Shanghai, China), Ni_0.8_Co_0.1_Mn_0.1_(OH)_2_ precursor (300 mesh, Zhejiang Power New Energy, 99.9%, China), lithium hydroxide monohydrate (LiOH·H_2_O, Aldrich, AR), and ethanol (CH_3_CH_2_OH, Zhiyuan, AR) were used as purchased without any further purification. Deionized water (DI) was homemade.

### 2.2. Assembly of MXene/PP Separator

MXene (Ti_3_C_2_T_x_) was prepared according to the works in [37,38]. Typically, 30 mL of concentrated HCl, 10 mL deionized water, and ~3.2 g lithium fluoride were taken and mixed well in a Teflon reactor. Then, ~1 g Ti_3_AlC_2_ was subsequently added into the mixed solution and stirred at 40 °C for 48 h in a water bath. After the reaction was deemed to be finished, the resulted solution was then centrifuged at 3500 rpm, and washed with deionized water until the pH of the supernatant reached neutral. The collected precipitate (MXene) was redispersed in 200 mL of deionized water and sonicated for 4 h with ice bath. The MXene dispersion was then centrifuged at 5000 rpm for 30 min to collect the upper solution, which belong to few-layer MXene dispersion (~1 mg/mL).

**Figure 1:**
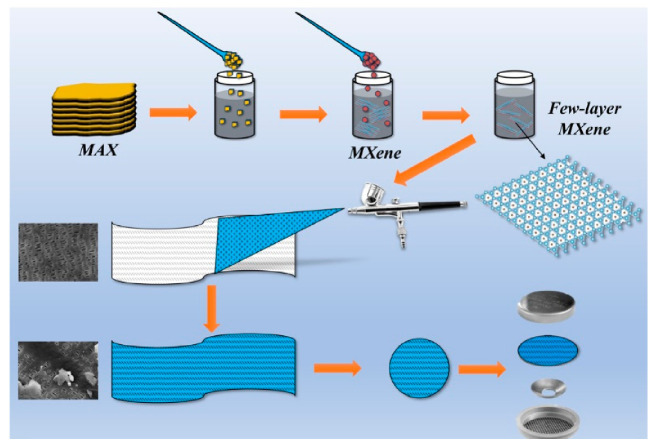
Schematic diagram of assembling MXene/PP diaphragm.

Then, 3 mL of the few-layer MXene dispersion was taken and sprayed uniformly on a PP diaphragm with a diameter of 16 mm by a spray gun (as shown in Figure 1). After air-drying for 2 h, the MXene/PP diaphragm was subsequently transferred to a vacuum oven and dried at 45 °C for 12 h. The MXene loading on each piece of PP diaphragm was measured to be ~0.5 mg/pcs.

### 2.3. Characterization Methods

A field emission scanning electron microscope (Hitach, SU8220, Tokyo, Japan) was used to observe the microscopic morphology of MXene, PP, and MXene/PP diaphragms, and the acceleration voltage was 10 kV. The composition and structure of the diaphragms were characterized by a Fourier infrared spectrometer (Thermofisher, Nicolet IS50, Waltham, MA, USA). One microliter of electrolyte (1 mol/L LiPF_6_ dissolved in DC/EC/DEC (1:1:1, *v*/*v*/*v*) was dropped on the surface of the diaphragms to test their contact angles by a tester (Chengde Jinhe Instrument Manufacturing Co., Ltd., Chengde, China).

### 2.4. Electrochemical Measurement

In order to study the enhancement of the novel MXene/PP separator on the NCM811 cathode, the 2032-coin cells of NCM811/Li were assembled in a glove-box filled with argon (Mikrouna, Shanghai, China). The NCM811 and pure Li metal were applied as cathode and anode, respectively. The PP (Celgard 2400) and MXene/PP were used as the separator. The amount of electrolyte (1 mol/L LiPF_6_ in EC/DEC/EMC, 1:1:1 by volume) was ~60 µL for each batch cell. In case of the cell by using MXene/PP separator, the NCM811 cathode was right next to MXene coating. The battery system (Neware, Shenzhen, China) is used to test electrochemical performances of NCM811/Li within a voltage range of 2.8~4.3 V vs. Li^+^/Li at room temperature. The cycle performance was tested at a current density of 0.5 C (1C = 200 mAh/g). The current density for rate performance gradually increased from 0.1 C to 16 C and then returned to 0.1 C. The constant current-constant voltage (CC-CV) charging mode was used for the electrochemical cycling, and the cut-off current of constant voltage charging was 4 mA/g (0.02 C). Cyclic voltammetry (CV) and electrochemical impedance spectroscopy (EIS) were measured at a scan rate of 0.1 mV/s and demonstrated from 100 kHz to 100 mHz with an amplitude of 5.0 mV via workstation (Chenhua, CHI760E, Shanghai, China), respectively.

## 3. Results and Discussion

**Figure 2:**
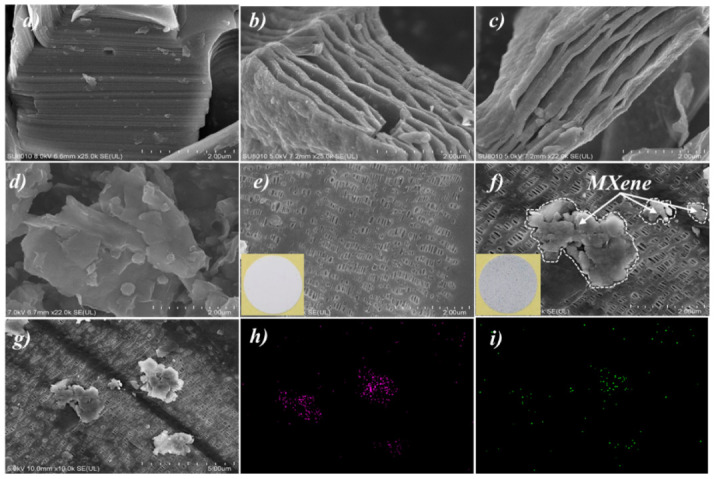
SEM images of (**a**) Ti_3_AlC_2_, (**b**–**d**) MXene, (**e**) PP, (**f**,**g**) MXene/PP separator, and EDS analyses of (**h**) Ti and (**i**) O elements.

Figure 2a shows the SEM images for Ti_3_AlC_2_ (MAX) morphology, which displayed the special characteristic of close-packed multilayer structures [24,32,39]. Figure 2b,c demonstrates the morphology of the etched MXene. The layers between the MXene were “open” with accordion-like characteristics [17,23,35]. However, the MXene obtained at this state was at the micron-level, which was not suitable for being coated on the PP to prepare MXene/PP hybrid separator. Therefore, the blockbuster and multilayered MXene should be further broken to obtain the few-layer ones. The morphology of the fewer-layer MXene is displayed in Figure 2d. The accordion-like MXene was destroyed macroscopically, forming a single-layer or few-layer structure. Due to a relatively large specific surface area, the single-layer and/or few-layer MXene were agglomerated together. Note that the agglomeration would not affect the microscopic layered structure of MXene (nano-level). Figure 2e–g demonstrates the morphologies of PP and MXene/PP separator, respectively. Obviously, the PP separator delivered many tiny holes for Li ions transportation on the surface. Moreover, the insets of Figure 2e,f display the digital photos of the PP and MXene/PP. After being coated with MXene, the MXene/PP changed its appearance from the white of PP to gray. However, the MXene/PP separator still retained a porous structure with a little hole being covered by MXene. The results indicated that the MXene should be loaded on the surface of PP without destroying its original pore structure. Figure 2h, i display the EDS results of Ti and O elements of the MXene/PP separator, which were the main components of MXene on the separator. Associating with Figure 2g, the bright and contrasting substances on the film were detected to contain Ti and O elements. Moreover, Ti/O elements were also detected in some places where the contrast of MXene was not particularly obvious, indicating that there was still single layer MXene without agglomeration on the MXene/PP separator.

**Figure 3:**
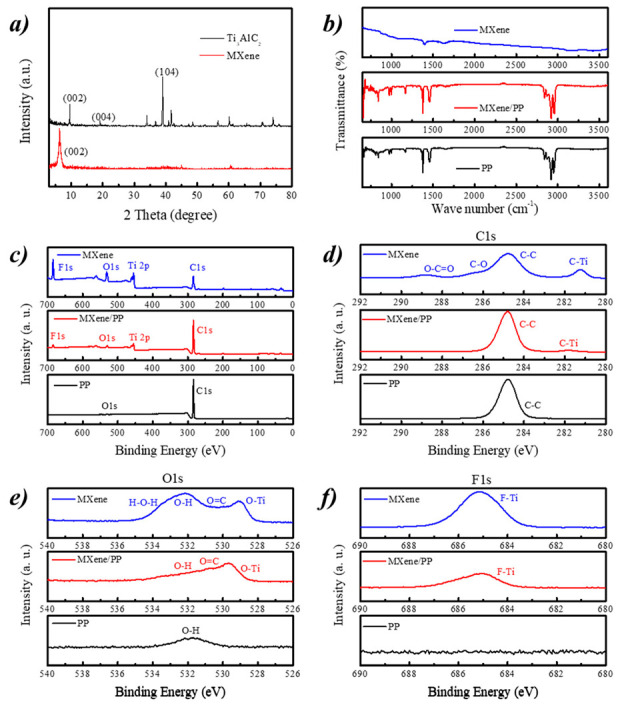
(**a**) XRD patterns of MAX and MXene; (**b**) FT-IR spectra; and (**c**) XPS survey and high resolution of (**d**) C1s, (**e**) O1s, and (**f**) F1s for MXene, PP, and MXene/PP samples.

Figure 3a shows the XRD patterns of Ti_3_AlC_2_ and MXene. The Ti_3_AlC_2_ sample displayed three characteristic peaks of (002), (004), and (104) planes at ~9.52°, ~19.08°, and ~38.96°, respectively. After etching, the attained MXene almost observed a single relative strong peak associating to the (002) plane. Moreover, the single peak of (002) shifted from ~9.52° of Ti_3_AlC_2_ to ~6.26° of MXene. Therefore, the planar spacing of (002) in MXene was measured to be 1.411 nm. Figure 3b displays the FT-IR spectra of MXene, PP, and the MXene/PP separator. As seen from the FT-IR results, the peaks at ~2839, ~1377, and ~841 cm^−1^ were attributed to the stretching vibration of methyl and methylene, methyl bending, and the absorption of stereo crystalline state of ordinary PP separator, respectively [40]. After the MXene was loaded on the PP, the obtained MXene/PP separator showed litter changes. Moreover, as the several characteristic peaks of MXene were overlapped with PP, they could not be distinguished in MXene/PP. Therefore, it indicated that the MXene material should show a small effect on the molecular chain structure of the PP separator and be electrostatically adsorbed on its surface. Note that, the peak strength of the MXene/PP separator became weak by comparing with the PP, which may be caused by the interference of MXene on the surface.

Figure 3c–f shows the XPS survey and high resolution of C1s, O1s, and F1s for MXene, PP, and MXene/PP samples, respectively. The survey of PP separator demonstrated a peak of C1s and a weak of O1s from the environment (Figure 3c). The MXene/PP and MXene delivered four strong peaks associating to C1s, Ti 2p, O1s, and F1s, which indicated that the MXene was successfully loaded on the MXene/PP [27,41,42]. Furthermore, from the high resolution of C1s (Figure 3d), an obvious peak at ~281.8 eV of C–Ti bond could be detected in MXene/PP separator [20,24]. Moreover, the O-Ti (Figure 3e) and F-Ti (Figure 3f) peaks were also found in the O1s and F1s high resolution spectra of MXene/PP respectively, which were not observed in PP sample. Note, the O1s of MXene/PP was different from that of MXene, suggesting that the –O and/or –OH functional groups should be the main link factors between MXene and PP.

**Figure 4:**
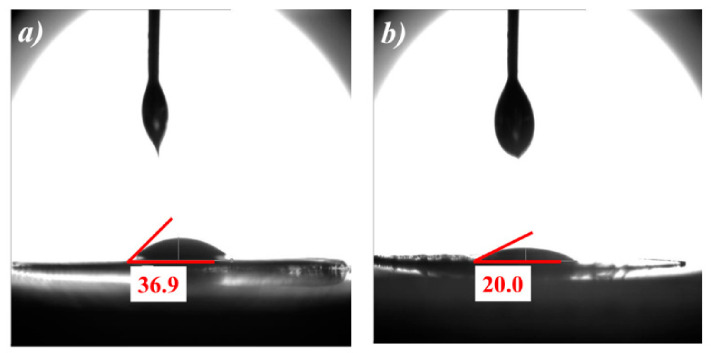
Contact angles of (**a**) PP and (**b**) MXene/PP separators.

Figure 4 demonstrates the contact angle test of ordinary PP and MXene/PP separators. The liquid for the test was the common electrolyte of lithium ion battery (1 mol/L LiPF_6_ + DC/EC/DEC (*V*:*V*:*V* = 1:1:1). It can be seen that the PP film showed poor wettability to the electrolyte, and its contact angle was ~36.9°. After being loaded MXene, the MXene/PP separator was significantly reduced to ~20.0°, indicating that the wetting of the electrolyte on the separator was improved. Moreover, the enhancement should absolutely be attributed to the MXene on the surface, which included abundant electrophilic functional groups such as –OH, –O, and –F.

**Figure 5:**
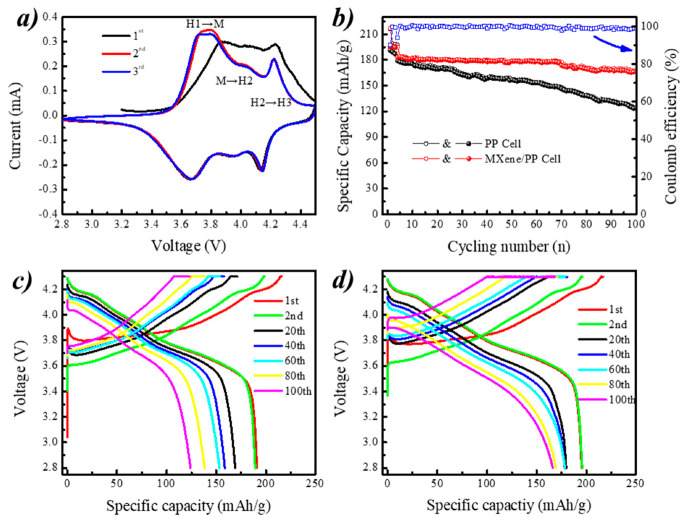
(**a**) CV curve of MXene/PP cell and (**b**–**d**) cycling performances of PP and MXene/PP cells.

In order to explore the effect of MXene/PP on the Ni-rich cathode, the NCM811 material and the ordinary PP (or MXene/PP) separator have been used as the positive electrode and separator, respectively, to assemble a Li/NCM811 cells for characterizing its electrochemical performances. Figure 5a shows the CV curve of MXene/PP cell at scanning rate of 0.1 mV/s between 2.8 and 4.5 V. The MXene/PP cell performs three peak couples related to phase transition reactions of H1↔M (hexagonal to monoclinic), M↔H2 (monoclinic to hexagonal), and H2↔H3 (hexagonal to hexagonal). Note that the peak related to M↔H2 became weak, which was corresponding to the previous work [36]. Figure 5b displays the cycling performance of the coin cells assembled with PP and an MXene/PP (shorten as PP and MXene/PP cell from here) separator at a current density of 0.5 C. After 100 cycles, the coulombic efficiency of MXene/PP cell was still close to a relatively high value of almost ~100% with little change. Moreover, the MXene/PP cell demonstrated more stable with a specific discharge capacity of 166.2 mAh/g at the 100th cycle from the initial 183.2 mAh/g to with a higher capacity retention of ~90.7%. By comparing with the MXene/PP cell, the PP cell showed a significant downward trend from the initial 179.0 to 123.2 mAh/g of 100th cycle with a lower retention of ~68.8%. Obviously, the MXene/PP displayed greatly effects on the cycle stability of NCM811 cathode. According to our previous work [35,36], the cycling improvement of MXene/PP cell should be assigned to the MXene attached on the surface, which can offer more deformation space for the volume expansion of NCM811 during the charging–discharging process and prohibits the irreversible Ni/Co/Mn elements migration via absorption process [36].

Figure 5c displays the voltage–capacity curves of PP cell at 0.5 C cycling. The first charge and discharge specific capacities of PP cell were 215.9 and 191.1 mAh/g (0.1 C) with a lower First Efficiency (FE) of 88.5%. As the cycle progressing, the discharge capacities at the 20th, 40th, 60th, 80th, and 100th cycle were 168.9, 158.6, 153.2, 138.3, and 124.1 mAh/g, respectively. Figure 5d shows the voltage–capacity curves of the MXene/PP cell under the 0.5 current density cycle. It can be seen that the specific capacity for the MXene/PP cell was 217.0 and 195.3 mAh/g (FE = 90.0%) during the first charge and discharge, respectively. Compared with ordinary PP cell, the MXene/PP cell showed enhancement on the discharge capacity and First Efficiency. Considering the cells belonging to constant current and constant voltage mode (CC-CV), we suggest that the extra capacity of NCM811 in the MXene/PP cell should be attributed to the intercalation/store of lithium ions between the layered MXene on the surface of PP [36]. As discussed before, the MXene/PP discharge capacities at the 20th, 40th, 60th, 80th, and 100th cycle were 179.7, 179.2, 179.5, 169.3, and 166.2 mAh/g, respectively. Note that during the first 70 cycles, the discharge capacities of MXene/PP cell showed hardly drops. Therefore, it can be inferred that the PP separator loaded with MXene can significantly improve the long cycle performance of the nickel-rich NCM811 material.

**Figure 6:**
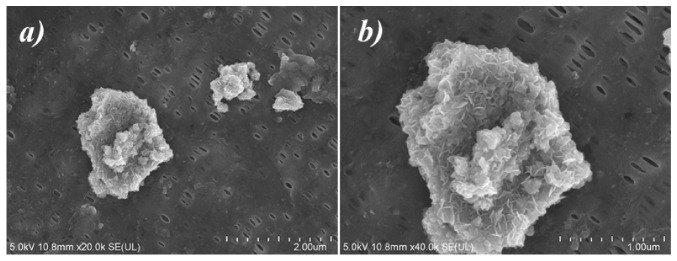
SEM images of MXene/PP separator after cycling: (**a**) 20,000× and (**b**) 40,000×.

Figure 6 displays the SEM images of MXene/PP separator after long-term cycling from disassembled coin cell. The holes for Li-ion transportation on the PP diaphragm were still intact, and the MXene loaded on the PP had not been peeled off or dissolved in the electrolyte (Figure 6a). However, the microscopic appearance of the loaded MXene had been changed. Before the cycle, MXene had a few-layer and single-layer structure (as shown in Figure 2f). After cycling, the surface of MXene presented a porous structure as displayed in the Figure 6b. The formation of the porous structure should be assigned to the corrosion of trace hydrofluoric acid in electrolyte and electric field during cycling. The porous structure of MXene obtained by corrosion was more conducive to adsorbing the high-valent metal ions of NCM811 (Ni/Co/Mn) and accelerating the migration of Li, thereby improving the stability of the battery.

**Figure 7:**
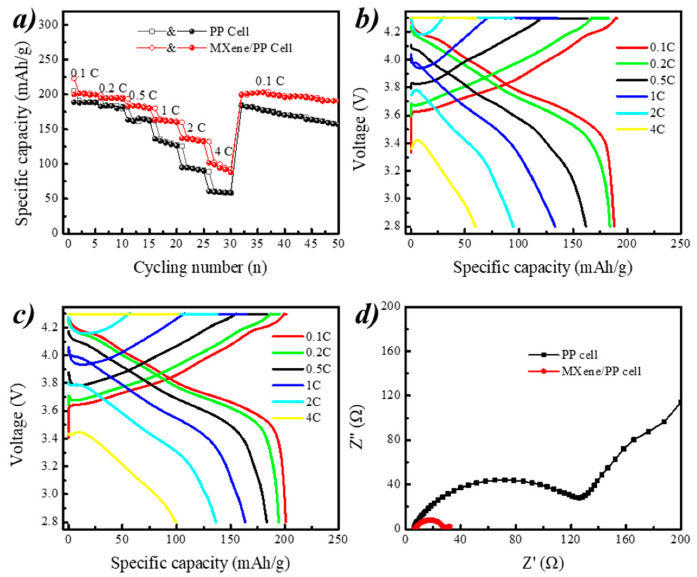
(**a**–**c**) Rate performances and (**d**) electrochemical impedance spectroscopy (EIS) spectra of PP and MXene/PP cells.

Figure 7a displays the rate performances of the ordinary PP and MXene/PP cells. It can be obviously seen that the specific discharge capacities of the MXene/PP cell were higher than that of the PP cell at different rates. Even at 2 C current density, the capacity of MXene/PP remained at ~136 mAh/g, which was higher than that of PP battery at 1 C current density of ~133 mAh/g. After larger rates cycling, the MXene/PP cell could still return to the original discharge specific capacity of 198.9 mAh/g with recovery efficiency of ~99.5% while the PP cell remained 184.6 mAh/g (~97.7% resilience) when they came back to the 0.1 C cycling. Moreover, the MXene/PP cell still maintained a certain stability during the subsequent charging and discharging process. This may be attributed to two-dimensional structure channel of MXene material, which could accelerate ion transmission and MXene improve the stability of the ternary NCM811 material [36].

Figure 7b,c demonstrates the voltage–capacity curves of the common PP and MXene/PP cells during charge and discharge at current densities of 0.1 C, 0.2 C, 0.5 C, 1 C, 2 C, and 4 C, respectively. The specific discharge capacities of PP cell were 188.1, 184.1, 161.6, 133.2, 94.7, and 59.8 mAh/g while that of MXene/PP cell were 201.1, 194.5, 183.4, 163.3, 136.4, and 99.3 mAh/g, respectively. The average voltage platforms of MXene/PP cell were higher than that of PP cell. Meanwhile, the initial voltage drops (IR drops) of MXene/PP cell at first discharge process during rate cycling were lower than that of PP cell, indicating that the MXene/PP cell’s resistance to lithium ions was smaller. As MXene can store lithium ions between its layers leading to a shorter transport path of Li^+^, MXene/PP therefore obtained a better rate performance. The extra stored Li ions can be released to increase the actual specific capacity of NCM811 during the discharge process. Moreover, the MXene loaded on the PP separator included abundant functional groups, which was better for the wettability of PP with the electrolyte and could accelerate Li ions transmission. Therefore, MXene/PP cell showed better electrochemical performances than PP cell in both capacity and capacity retention during rate tests [7,11,12,36].

Figure 7d shows the electrochemical impedance spectra of MXene/PP cells, which were all consist of two parts. The first part was the fixed internal resistance (R_b_) of the cell in the low frequency region, which was the intersection of the semicircle in the high frequency region and the real axis. The R_b_ of MXene/PP and PP cells were measured to be ~6.2 and ~6.9 Ω, indicating that the consistency of cells assembly was relatively higher. The second part was a charge transfer impedance (R_ct_) and corresponded to a semicircular portion in the high frequency region. The R_ct_ of MXene/PP cells were ~28.0 Ω, suggesting that the charge transfer resistance of MXene/PP was smaller. All results were well consistent with the former electrochemical performances in long-term cycling and rate tests.

**Figure 8:**
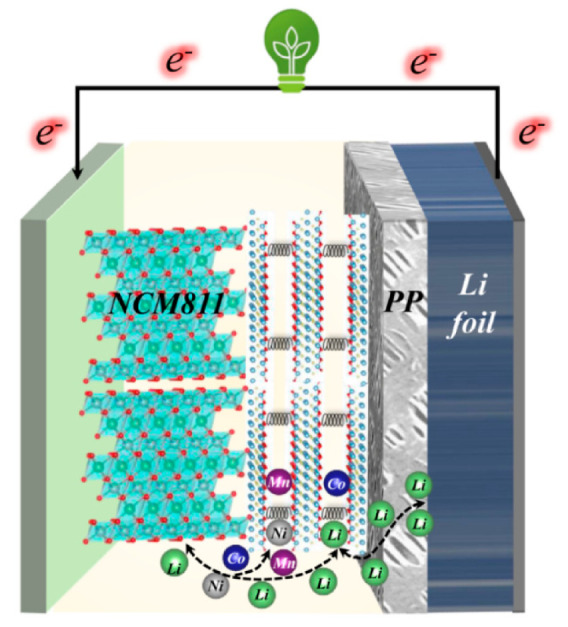
Schematic diagram of the mechanism of MXene/PP to enhance the NCM811 performances.

Figure 8 demonstrates the schematic diagram of the mechanism of MXene/PP to enhancing the NCM811 performance. By combining previous research results and existing data analysis [35,36,43,44,45,46,47], we attribute the enhancements of NCM811 electrochemical properties to the MXene on the PP surface as follows. (1) Flexible MXene with changeable layer spacing can be used to adjust the volume change of NCM811 during charge and discharge, thereby improving its cycle stability. (2) The MXene can store/release extra Li ions between its layers, which can increase the actual specific capacity of NCM811 and shorten the ionic diffusion distance. Moreover, (3) the MXene can prevent the transition metal (Ni/Co/Mn) from migrating to the negative electrode to form dendrites through adsorption, thus effectively reducing the risk of NCM811 material failure. Therefore, the MXene/PP separator delivers excellent performance in the application for Ni-rich NCM811 cathode.

## 4. Conclusions

In summary, a simple and effective method was used to coat the prepared few-layered MXene on the commercial PP separator and thereafter obtain MXene/PP hybrid membrane. The prepared few-layered MXene was first characterized and confirmed by XRD and SEM analyses. The micromorphology, structure, and wettability of the PP and MXene/PP separators were measured by SEM, FT-IR, and contact angle tests, respectively. The SEM analysis showed that the MXene was loaded on the surface of PP without destroying its original pore structure. The FT-IR results indicated that the loaded MXene show a certain influence on the surface condition of PP, which was corresponding to the wettability change from 36.9° of PP to 20.0° of MXene/PP confirmed by the contact angle test. Using the obtained MXene/PP as separator to assembly Li/NCM811 cells, we found that the MXene loaded PP showed great influence on the electrochemical performances of the NCM811 cathode. The Li/NCM811 cell with MXene/PP separator demonstrated more stable with a specific discharge capacity of 166.2 mAh/g at the 100th cycle from the initial 183.2 mAh/g to with a higher capacity retention of ~90.7% during 0.5 C current density cycling. After 100 cycles, the coulombic efficiency of MXene/PP cell was still close to a relatively high value of almost ~100% with little change. The specific discharge capacities of MXene/PP cell were 201.1, 194.5, 183.4, 163.3, 136.4, 99.3 mAh/g at current densities of 0.1 C, 0.2 C, 0.5 C, 1 C, 2 C, and 4 C, respectively. The Rct of MXene/PP cell was measured to be ~28.0 Ω, which was only a quarter of the PP cell (~125.9 Ω). All improvements were assigned to the sputtered MXene on the surface of PP separator, which could be used to adjust the volume change of NCM811 to improve its cycle stability, store/release extra Li ions to increase the actual specific capacity, shorten the ionic diffusion distance and prevent the transition metal (Ni/Co/Mn) from migrating to form dendrites through adsorption. The strategy to prepare MXene/PP separator by spraying the MXene on commercial PP is a facile, convenient and effective way to improve the electrochemical performance of NCM811, which is expected to achieve large-scale in high-performance lithium-ion batteries in the near future.
